# Heavy Metals in Water and Sediment: A Case Study of Tembi River

**DOI:** 10.1155/2014/858720

**Published:** 2014-01-29

**Authors:** Saeed Shanbehzadeh, Marzieh Vahid Dastjerdi, Akbar Hassanzadeh, Toba Kiyanizadeh

**Affiliations:** ^1^Health Center of Masjed Soleman, 8 Bangle Avenue, Masjed Soleman, Iran; ^2^Environment Research Center, School of Health, Isfahan University of Medical Sciences, Hezar Jerib Avenue, Isfahan, Iran; ^3^Faculty of Health, Isfahan University of Medical Sciences, Hezar Jerib Avenue, Isfahan, Iran

## Abstract

This study was carried out to examine heavy metals concentration in water and sediment of upstream and downstream of the entry of the sewage to the Tembi River, Iran. Samples were collected from upstream and downstream and were analyzed for Cd, Cr, Cu, Fe, Pb, Ni, and Zn by atomic absorption spectrophotometer. The results indicated that the average concentration of the metals in water and sediment on downstream was more than that of upstream. The comparison of the mean concentrations of heavy metals in water of the Tembi River with drinking water standards and those in the water used for agriculture suggests that the mean concentration of Cu and Zn lies within the standard range for drinking water and the mean concentration of Mn, Zn, and Pb lies within the standard range of agricultural water. The highest average concentration on downstream for Pb in water and for Mn in sediment was 1.95 and 820.5 ppm, respectively. Also, the lowest average concentration on upstream was identified for Cd in water and sediment 0.07 and 10 ppm, respectively. With regard to the results, it gets clear that using the water for recreational purposes, washing, and fishing is detrimental to human health and the environment.

## 1. Introduction

The entry of contaminants into the environment due to human and natural activities is one of the most important issues facing today's communities. Due to the industrial and economic growth and the production of a variety of compounds and chemicals followed by increased consumption man makes some unwanted pollutants, many of which cause serious problems and risks for the environment and for man himself. The most important natural resources of environmental pollution are soil and rock weathering and natural events such as earthquakes and floods [1]. The entry of municipal, industrial, and agricultural waste into the environment is another way of the environment pollution by human. Water resources are among the most critical resources. The importance of water resources, particularly surface waters (rivers), in meeting the water needs of humans, animals and industries indicates the essential need to protect them against contamination. As municipal, industrial, and agricultural waste enters the water, biological and chemical contaminants including heavy metals also enter water resources. Although some of these metals are essential as micronutrients, their high concentration in the food chain can cause toxicity and environmental impacts and endanger aquatic ecosystems and their users [2, 3]. Rivers as water resources, not only in terms of drinking water supply but also in terms of their function in recreational and sport activities such as water sports and fishing, are very important to man's health. The investigation of the entry sources, effects, and control of pollutants in rivers has, therefore, always been one of the research topics of environmental scientists. Tembi River, located in the outskirts of Masjed Soleyman, Iran, is open to untreated municipal sewage, runoff, abattoir wastewater, and leachate of solid wastes around it [4]. Regarding the above-mentioned issues and due to the fact that it is a place for the migration of birds in cold seasons as well as for public promenade, swimming, and fishing the present study was thus carried out to investigate the concentration of heavy metals such as copper, zinc, lead, chromium, cadmium, nickel, and manganese due to the discharge of sewage of Masjed Soleyman into this river.

## 2. Materials and Methods

### 2.1. Sample Collection

After determining the location of the sampling points before and after the entry of the sewage, 96 samples (48 water samples and 48 sediment samples) were collected by grab method for 1-year period; four samples of water and four samples of sediment were collected in each month EE and 3 liters of water was collected in each of sampling from below the water surface and 30 g of sediment samples from a depth of 20 cm under the bed river were collected [5]. Samples of water acidified with nitric acid (PH = 2) along with other samples were transferred to the laboratory.

### 2.2. Sediment Samples Preparation

30 g of sediment samples was dried at 105°C. One gram of each sample was weighed carefully and 5 mL of concentrated nitric acid (Merck, 99.99%) was added to each sample. The sample, then, was heated up to 80°C until near dryness. The addition of acid and the process of heating were repeated two more times [6]. An amount of water was added to the residual material. The suspension was filtered (Whatman filter Merck, 0.45 *μ*m) and the filtrate was diluted by deionized water to a final volume of 50 mL.

### 2.3. Water Samples Preparation

Three liters of each collected water sample was first concentrated on a sandy oven at 80°C until the volume reached 50 mL. Then 4 mL concentrated sulfuric acid (Merck, 98%) was added to each sample and Digested by digesdahl apparatus for 3 minutes. The 10 mL hydrogen peroxide (Merck, 30%) was then added and heated until oxidation was completed. After cooling, each sample filtered by filter (Whatman filter Merck, 0.45 *μ*m). The filtrate was diluted by deionized water to a final volume of 50 mL.

### 2.4. Samples Analysis

The prepared samples were analyzed by a Graphite furnace atomic absorption spectrometry (GFAAS, Model AAnalyst300) to determine the metals [6].

### 2.5. Statistical Analysis (Analysis of Variance, *t*-Test, and Least Significant Differences Test That Means “LSD”)

It was conducted to determine the difference of metal ion concentrations among the water and sediment in upstream and downstream of Tembi River in different seasons. A *P* value ≤ 0.05 was considered significant. The correlation analysis was also carried out to determine the relationship between the concentration of metal ions in water and sediment.

## 3. Results 

The mean concentration of Cd, Cr, Cu, Fe, Pb, Nis and Zn in water and sediment samples before and after the sewage entry into the Tembi River is presented with their standard deviations in Tables 1 and 2.

## 4. Discussion

The results of the analysis of water and sediment samples in upstream and downstream of the river revealed that the mean concentration of Cd, Cr, Cu, Fe Mn, Pb, and Zn in the downstream water was higher than that in the upstream water in different seasons (*P* < 0.001).  Table 1 indicates the effect of the entry of sewage (including untreated municipal sewage, runoff, abattoir wastewater, and leachate of solid wastes around the river) on Tembi River. In a similar research Wogu and Okaka [7] investigated 9 similar metals in the Warri River in Nigeria, which receives the industrial, agricultural, and urban sewage, and showed that the concentration of Cd, Cr, Mn, and Ni in this river is higher than the stated standard level for it, and that its water is harmful to the public health and hygiene [7].

It is to be noted that the mean concentration of metals in water in downstream and upstream has increased from Summer 2011 to Spring 2012. Pb has had the highest mean concentration of 1.95 ± 0.09 mg/L in downstream and Cd has had the lowest concentration of 0.07 ± 0.001 mg/L in upstream area. The seasonal distribution of the mean concentrations of metal of Cd, Cr, Cu, Fe, Mn, Ni, Pb, and Zn in Tembi River water have been shown to be as follows (the maximum amounts are related to hot seasons):

(1) (0.07–0.35,0.13–0.58,0.26–0.71,0.24–1.87,  0.07–0.91,0.16–0.8,0.59–1.91,0.12–0.4)mg/L.

These results show the effect of dry seasons and water evaporation on concentration increase of heavy metals in water. These results were similar to results of Obasohan and Eguavoen and Ahmed et al. [8] researches [8, 9]. Obasohan and Eguavoen [9] have stated in their research that dry seasons affect the accumulation of heavy metals in water and its reared fish. In a similar study, Ahmed et al. [8] investigated the distribution of heavy metals concentration in the Buriganga River in Nigeria and found out that the seasonal distribution of Cd, Cr, Cu, Ni, and Pb varies seasonally as follows:

(2) (7.08–12.33,489.27–645,107.38–201.29,  7.5–10.32,58.7–72.45)mg/L.

They have also stated that the maximum and minimum concentrations are those of Ni and Cd [8], respectively.

The comparison of the mean concentrations of heavy metals in the Tembi River water with the standard value in drinking water [10] and those in the water used for agriculture [11] for aquatic life [12] and surface water standards [13] suggests that the mean concentration of Cu lies within the standard range for drinking water and the mean concentrations of Mn and Cd lie within the standard range of agricultural water. Only do the mean concentrations of Zn lie within the standard range for all three kinds of water. In general, Tembi River water is not suitable for drinking and aquatic life. Mean concentrations of Cd, Cr, Mn, and Pb were higher than surface water standards (Table 3).

The mean concentrations of metals in the sediments of the Tembi River downstream have increased in comparison with those in its upstream. The maximum and minimum levels of concentration are related to Mn in downstream and Cd in upstream areas, respectively (Table 2). Regarding the rise in the concentrations of these metals in downstream water, the higher concentrations in downstream sediment are very reasonable. In fact; increased heavy metals concentration in water in downstream lead to increasing of their concentration in sediment in downstream. In a similar study Kowsari and Naei [14] stated that physicochemical characteristics of water affect the precipitation of these elements in sediment.

The mean concentration of metals in the sediments of the river upstream, where the sewage enters Tembi River, changes similarly in four seasons. In other words, the average concentration of metals in sediments in Autumn and Winter is lower than that in Summer. These amounts rise again when Summer starts. The amounts of heavy metals in sediment varied seasonally as follows:

 Summer > Autumn > Winter > Spring.

The cause of these changes was high rain in Autumn and Winter which gives rise to flow of the water in the river. Due to the turbulence created by increase of flow, some sediments and heavy metals inside them are displaced and carried away from the river bed. As Summer starts, the rise in temperature and evaporation and the end of the rain period cause the rise in heavy metals concentration in water and finally in sediments because metal ions transfer from water to sediment. The seasonal variation of distribution of Cd, Cr, Cu, Fe, Mn, Ni, Pb, and Zn in the sediments is as follows:

(3) (10–40,11–74,37–100,210–280,278–820,52–150,641–270,21–74)mg/kg.

In this regard Mor et al. [18], in a survey of pollution that resulted from oil wells and heavy metals in the Tembi River sediments, announced the amounts of variation in the concentrations of Cd, Cr, Cu, Ni, Pb, and Zn as follows:

(4) (2.39–6.5,70–130,16–74,11–33,56–120,50–236)mg/kg.

Also, Ahmed et al. [8] announced the seasonal distribution of the concentrations of Cd, Cr, Cu, Ni, and Pb in the sediments of the Buriganga River as follows:

(5) (2.36–4.25,118.63–218.19,21.75–32.54,147.06–258.17,64.71–77.13)mg/kg.

The comparison of mean concentrations of the these metals with the standards for fresh water sediments indicates that, except Zn, the mean concentrations of Cd, Cr, Cu, Ni, and Pb were higher than the standards for fresh water sediments [15, 16] while only the mean concentrations of Cd in marine sediments [17] were higher than the standards (Table 4). However, due to increased metals concentration in the sediments of Tembi River, the rise of concentrations of heavy metals, in comparison with guideline in sediments, is expected in future.

## 5. Concluding Remarks

Based on the above-mentioned points, it is clear that the water and sediments of the Tembi River were contaminated by heavy metals and, therefore, using this water for recreational purposes, washing, and fishing is detrimental to human health and the environment. It is, thus, necessary to take serious and essential measures to control the entry of the sewage, treat it before entering the river, manage the quality of water and the sediments of the river, and utilize water for various purposes.

## Figures and Tables

**Table 1:** Variations of average of heavy metals concentration in Tembi River water in different seasons (mg/L).

Heavy metals	Sampling point	Sampling time			
		Summer 2011	Autumn 2011	Winter 2011	Spring 2012
Cd	1	0.07 ± 0.010	0.12 ± 0.700	0.24 ± 0.002	0.25 ± 0.030
	2	0.1 ± 0.006	0.26 ± 0.010	0.30 ± 0.010	0.35 ± 0.050
Cr	1	0.13 ± 0.010	0.29 ± 0.005	0.16 ± 0.010	0.17 ± 0.010
	2	0.25 ± 0.010	0.40 ± 0.05	0.31 ± 0.060	0.58 ± 0.005
Cu	1	0.26 ± 0.020	0.45 ± 0.080	0.56 ± 0.010	0.62 ± 0.010
	2	0.40 ± 0.01	0.54 ± 0.050	0.63 ± 0.010	0.71 ± 0.010
Fe	1	0.24 ± 0.010	0.18 ± 0.010	0.82 ± 0.040	1.1 ± 0.005
	2	0.26 ± 0.020	0.28 ± 0.030	1.1 ± 0.050	1.87 ± 0.030
Mn	1	0.07 ± 0.003	0.09 ± 0.001	0.81 ± 0.01	0.82 ± 0.010
	2	1.00 ± 0.007	1.25 ± 0.030	1.40 ± 0.020	0.91 ± 0.020
Ni	1	0.16 ± 0.01	0.40 ± 0.050	0.52 ± 0.010	0.83 ± 0.02
	2	0.50 ± 0.050	0.75 ± 0.010	0.65 ± 0.070	0.80 ± 0.020
Pb	1	0.59 ± 0.06	1.45 ± 0.030	0.81 ± 0.080	1.64 ± 0.020
	2	1.80 ± 0.070	0.85 ± 0.070	1.02 ± 0.010	1.91 ± 0.030
Zn	1	0.12 ± 0.000	0.23 ± 0.005	0.34 ± 0.000	0.11 ± 0.000
	2	0.20 ± 0.010	0.29 ± 0.005	0.51 ± 0.020	0.40 ± 0.000

1: upstream; 2: downstream.

**Table 2:** Variations of average of heavy metals concentration in Tembi River sediments in different seasons (mg/kg).

Heavy metals	Sampling point	Sampling time			
		Summer 2011	Autumn 2011	Winter 2011	Spring 2012
Cd	1	14 ± 0.7	10 ± 0.5	10 ± 0.5	24 ± 1.00
	2	40 ± 1.1	20 ± 1.5	14 ± 0.5	24 ± 0.5
Cr	1	57 ± 1.5	40 ± 0.5	28 ± 2.0	42 ± 1.1
	2	74 ± 0.5	70 ± 2.0	11 ± 1.0	67 ± 4.0
Cu	1	70 ± 1.1	50 ± 1.0	37 ± 1.5	49 ± 1.0
	2	100 ± 2.6	66 ± 1.0	40 ± 1.7	54 ± 1.0
Fe	1	250 ± 6.0	230 ± 1.0	220 ± 1.0	230 ± 0.5
	2	280 ± 3.0	234 ± 2.0	210 ± 0.5	233 ± 1.05
Mn	1	715 ± 1.00	310 ± 16	310 ± 11	302 ± 12
	2	820 ± 2.5	310 ± 5.0	278 ± 5.0	341 ± 9.0
Ni	1	107 ± 2.0	63 ± 3.2	52 ± 2.9	129 ± 11
	2	150 ± 3.6	80 ± 7.6	87 ± 2.5	143 ± 7.0
Pb	1	240 ± 3.4	198 ± 2.3	141 ± 4.0	151 ± 5.0
	2	270 ± 0.5	236 ± 2.0	178 ± 3.3	210 ± 2.0
Zn	1	54 ± 2.0	30 ± 1.5	24 ± 2.0	32 ± 1.0
	2	74 ± 2.0	30 ± 1.5	21 ± 2.0	38 ± 1.0

1: upstream; 2: downstream.

**Table 3:** Comparison of average of heavy metals concentration in upstream and downstream of Tembi River with guideline for water (mg/L) [10–13].

Heavy metals	Cd		Cr		Cu		Fe		Mn		Ni		Pb		Zn	
sampling point	1	2	1	2	1	2	1	2	1	2	1	2	1	2	1	2
Average	0.17	0.25	0.19	0.29	0.47	0.57	0.56	0.96	0.45	0.94	0.48	0.68	1.13	1.4	0.2	**0.35**
Drinking water standards	0.005		0.1		1.3		0.3		0.05		—		0.00		**5.00**	
Irrigation water standards	0.01		0.1		0.2		—		2.00		0.2		5.00		**2.00**	
Aquatic life standards	0.01		0.05		0.05		—		0.1		—		0.05		**<0.1**	
Surface water standards	0.01		0.16		—		—		0.1		0.144		0.005		—	

1: upstream; 2: downstream.

**Table 4:** Comparison of average of heavy metals concentration in upstream and downstream sediments with guideline for sediments (mg/kg) [15–17].

Heavy metals	Cd		Cr		Cu		Fe		Mn		Ni		Pb		Zn	
Sampling point	1	2	1	2	1	2	1	2	1	2	1	2	1	2	1	2
Average	14.5	24.5	42	55.5	51.5	65	232	239	409	437	87.8	115	182	223	35	**41**
Fresh water sediments guideline	0.99		43.4		31.6		—		—		22.7		35.8		**121**	
Marin sediments	5.1		260		390		—		—		—		450		**410**	

1: upstream; 2: downstream.
